# Hepatic glucose production rises with the histological severity of metabolic dysfunction-associated steatohepatitis

**DOI:** 10.1016/j.xcrm.2024.101820

**Published:** 2024-11-19

**Authors:** Silvia Sabatini, Partho Sen, Fabrizia Carli, Samantha Pezzica, Chiara Rosso, Erminia Lembo, Ornella Verrastro, Ann Daly, Olivier Govaere, Simon Cockell, Tuulia Hyötyläinen, Geltrude Mingrone, Elisabetta Bugianesi, Quentin M. Anstee, Matej Orešič, Amalia Gastaldelli

**Affiliations:** 1 Cardiometabolic Risk Unit, Institute of Clinical Physiology, CNR, 56121 Pisa, Italy; 2 Turku Bioscience Centre, University of Turku and Åbo Akademi University, 20520 Turku, Finland; 3 Department of Medical Sciences, Division of Gastro-Hepatology, A.O. Città della Salute e della Scienza di Torino, University of Turin, 10124 Turin, Italy; 4 Department of Medical and Surgical Sciences, Università Cattolica del Sacro Cuore, Rome, Italy; 5 Fondazione Policlinico Universitario Agostino Gemelli IRCCS, Rome, Italy; 6 Translational and Clinical Research Institute, Faculty of Medical Sciences, Newcastle University, Newcastle upon Tyne NE2 4HH, UK; 7 Department of Imaging and Pathology, KU Leuven and University Hospitals Leuven, Leuven, Belgium; 8 School of Science and Technology, Örebro University, 70281 Örebro, Sweden; 9 Newcastle NIHR Biomedical Research Centre, Newcastle upon Tyne Hospitals NHS Trust, Newcastle upon Tyne NE7 7DN, UK; 10 School of Medical Sciences, Örebro University, 70281 Örebro, Sweden; 11 Diabetes Division, The University of Texas Health Science Center at San Antonio, San Antonio, TX, USA; 12 Division of Diabetes & Nutritional Sciences, School of Cardiovascular and Metabolic Medicine & Sciences, King’s College Hospital, London, UK

**Keywords:** MASLD, MASH, NASH, type 2 diabetes, genome-scale metabolic modeling, fluxomics, hepatic glucose production, gluconeogenesis, insulin resistance, liver fibrosis

## Abstract

Metabolic dysfunction-associated steatotic liver disease (MASLD) and steatohepatitis (MASH) are associated with a high prevalence of type 2 diabetes (T2D). Individuals with MASLD exhibit insulin resistance (IR) and hyperglycemia, but it is unclear whether hepatic glucose production (HGP) is increased with MASLD severity. We evaluated HGP in a cohort of histologically characterized individuals with MASL/MASH using stable isotope infusion (6,6-^2^H_2_-glucose, U-^2^H_5_-glycerol) and liver-specific genome-scale metabolic models (GEMs). Tracer-measured HGP is increased with liver fibrosis and inflammation, but not steatosis, and is associated with lipolysis and IR. The GEM-derived gluconeogenesis is elevated due to high glucogenic/energy metabolite uptakes (lactate, glycerol, and free fatty acid [FFA]), and the expression of insulin action genes (IRS1, IRS2, and AKT2) is reduced in MASH with fibrosis F2–F4, with/without T2D, suggesting these as putative mechanisms for increased fasting HGP and hyperglycemia. In conclusion, elevated HGP, lipolysis, and IR help to explain the mechanisms for the increased risk of hyperglycemia and T2D in MASH.

## Highlights

• Glucose production is higher in individuals with MASH compared to simple steatosis
• Glucose production is higher with liver inflammation and fibrosis, not steatosis
• Hepatic and adipose insulin resistance contribute to glucose production in MASH
• The putative mechanism involves excess glucogenic/energy substrates to the liver

## Graphical abstract

**Figure:**
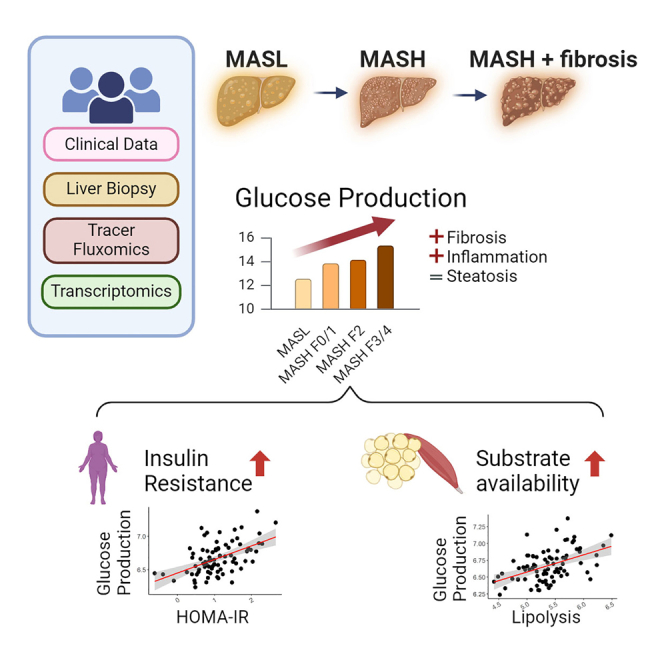

## Teaser

Sabatini et al. demonstrate that glucose production is enhanced in individuals with MASH and is associated with hepatic fibrosis and inflammation. These findings reveal altered hepatic glucose metabolism, driven by hepatic and extrahepatic insulin resistance that contributes to the increased risk of hyperglycemia and T2D in individuals with MASH.

## Introduction

Metabolic dysfunction-associated steatotic liver disease (MASLD), previously named non-alcoholic fatty liver disease, is defined as the excessive accumulation of triglycerides in the liver (steatotic liver disease) in the presence of at least one cardiometabolic risk factor and no other apparent causes for the condition^1^ and comprises a spectrum of diseases spanning from isolated steatosis (MASL) to steatohepatitis (MASH).^2^ Although MASLD is mainly related to altered lipid metabolism in both liver and adipose tissue,^3^^,^^4^ glucose metabolism is also impaired.^5^ MASLD is associated with an increased risk (hazard ratio 2.69) of developing type 2 diabetes (T2D)^6^ and conversely, the prevalence of MASLD is higher in T2D.^7^ Individuals with diabetes have also a high prevalence of advanced fibrosis and cirrhosis,^8^^,^^9^^,^^10^ which are the major risk factor for liver related outcomes,^8^ and the progression of fibrosis in these individuals is faster compared to those without T2D.^11^

Glucose metabolism has been studied mainly in subjects with MASLD, but only few studies were carried out in individuals with biopsy-proven MASH.^5^ In MASLD, fasting glucose production, which is mainly hepatic (HGP),^12^ has been found to be similar or increased compared to healthy controls.^3^^,^^5^^,^^13^^,^^14^ While it is known that individuals with MASLD display insulin resistance (IR) both in the liver and in peripheral organs (i.e., muscle and adipose tissue) even in the absence of obesity or diabetes,^3^^,^^13^^,^^14^ and that peripheral IR is increased with both steatosis^3^ and fibrosis,^15^ less is known about the impact of MASH phenotype (i.e., ballooning and inflammation) and fibrosis on hepatic glucose metabolism. Up to now, the investigation of hepatic glucose metabolism was done only in small groups of individuals with MASLD; few tracer studies have been conducted and only in small groups of subjects with biopsy-proven MASH.^5^ Moreover, the majority of these studies have focused on the relationship with steatosis, without investigating if hepatic glucose metabolism was altered with the severity of histology in subjects with biopsy-proven MASH independently of diabetes.^5^

Thus, the aim of this study was to examine how the transition from isolated steatosis (MASL) to MASH and the stage of fibrosis affect hepatic glucose metabolism in fasting condition, considering the impact of IR in the liver and peripheral organs such as muscle and adipose tissue, as well as of T2D. In this context, glucose production was measured in a large cohort of subjects with liver biopsy from the Elucidating Pathways of Steatohepatitis (EPoS) cohort using state-of-the-art technique, i.e., stable isotope tracer infusion; the intra-hepatic glucose metabolism was then explored by personalized genome-scale metabolic models (GEMs) constructed using individual liver transcriptomics and clinical data. These data provide information on the regulation of fasting hepatic glucose fluxes and metabolism in MASH considering also the separate impact of T2D.

## Results

### Description of the EPoS cohort

Data were generated during the H2020-EPoS project in which histologically characterized individuals with MASLD were recruited.^16^

Glucose production (HGP) and hepatic IR (Hep-IR) were measured by stable isotope tracer infusion in 80 subjects without diabetes whose clinical characteristics are shown in Table 1 (EPoS-flux group). Data were normalized by lean body mass (LBM) to take into account the differences in BMI (range from 18 to 54 kg/m^2^).^17^ This group included subjects spanning from (1) isolated steatosis (“MASL”), (2) MASH with fibrosis score 0 or 1 (“MASH-F0/F1”), (3) MASH with fibrosis score 2 (“MASH-F2”), and (4) MASH with fibrosis score 3 or 4 (“MASH-F3/F4”).

**Table 1:** Clinical characteristics of the EPoS-flux group

	*n*	MASL	MASH F0/F1	MASH F2	MASH F3/F4
*N*	80	17	31	15	17
Gender (m/f)	80	17/0	27/4	12/3	11/6∗
T2D (yes/no)	80	0/17	0/31	0/15	0/17
Age (years)	80	40.82 ± 2.52	38.65 ± 1.73	43.67 ± 3.95	44.24 ± 3.22
BMI (kg/m^2^)	80	27.95 ± 1.26	30.24 ± 1.65	32.03 ± 1.89	29.41 ± 0.91
ALT (U/L)	80	69.41 ± 10.59	69.06 ± 5.91	54.13 ± 9.85	96.35 ± 9.36∗°^∧^
AST (U/L)	79	36.71 ± 4.37	35 ± 2.08	32 ± 3.12	51.47 ± 3.96∗°^∧^
Glucose (mg/dL)	80	93.83 ± 1.76	94.73 ± 1.6	93.95 ± 3.5	100.87 ± 3.41
Insulin (mU/L)	80	11.73 ± 1.68	15.23 ± 2.1	14.65 ± 1.63	17.36 ± 2.06∗

Subjects with F4 *n* = 2. Cutoff for obesity: BMI 30 kg/m^2^. Mann-Whitney’s test *p* value: ∗ vs. MASL <0.05, ° vs. MASH-F01 < 0.05, ^∧^ vs. MASH-F2<0.05.

Intra-hepatic glucose metabolism was then explored in a larger number of subjects (*n* = 206, EPoS-transcriptomics group) by investigating personalized GEMs based on liver transcriptomics and clinical data (see STAR methods). The individuals of the EPoS-transcriptomics group were histologically characterized for MASLD with liver biopsy,^16^^,^^18^ had BMI range similar to the EPoS-flux group (from 20 to 45 kg/m^2^), but also comprised subjects without/with a previous diagnosis of T2D (54%) (Table 2). In 12 individuals, glucose fluxes were measured by both tracer infusion and GEMs (Figure S1A).

**Table 2:** Clinical characteristics of the EPoS-transcriptomics group

	*n*	NO T2D				T2D				*p* value
		MASL	MASH F0/F1	MASH F2	MASH F3/F4	MASL	MASH F0/F1	MASH F2	MASH F3/F4	
*N*	206	36	20	26	15	13	13	30	53	–
Gender (m/f)	206	26/10	12/8	14/12	11/4	8/5	10/3	15/15	27/26	–
Age	206	48.75 ± 2.18	50.55 ± 2.18	48.42 ± 2.9	57.6 ± 2.7∗°^∧^	59.15 ± 2.9	53.46 ± 3.97	55.37 ± 1.77	58.77 ± 1.18	§
BMI (kg/m^2^)	206	28.98 ± 0.72	30.43 ± 1.3	32.11 ± 1.12∗	30.52 ± 1.19	30.04 ± 1.17	29.42 ± 1.26	32.46 ± 0.79	33.34 ± 0.68∗°	§
ALT (U/L)	202	54.03 ± 6.36	71.55 ± 8.54∗	84.29 ± 9.42∗	79.53 ± 11.36∗	40.31 ± 4.04	57.85 ± 7.36	57.07 ± 4.61∗	69.43 ± 4.46∗	–
AST (U/L)	194	37.31 ± 2.61	33.47 ± 2.26	46.54 ± 4.47	47.67 ± 5.27°	29.67 ± 3.08	34.38 ± 1.92	41.64 ± 3.46∗	51.37 ± 3.02∗°^∧^	–
Glucose (mg/dL)	117	95.21 ± 2.57	91.26 ± 3.28	93.8 ± 2.77	114.37 ± 11.64°	149.02 ± 22.26	130.34 ± 10.44	145.79 ± 12.11	128.25 ± 5.37	§
Insulin (mU/L)	77	10.09 ± 1.37	17.48 ± 3.97	18.56 ± 3.05∗	20.72 ± 3.82∗	21.5 ± 7.82	21.1 ± 6.03	23.37 ± 5	23.97 ± 3.66	§
TG	110	1.62 ± 0.25	1.81 ± 0.59	1.36 ± 0.11	2 ± 0.38	3.5 ± 1.24	1.82 ± 0.22	1.74 ± 0.18	2.11 ± 0.23	§

Subjects with F4 *n* = 14. Mann-Whitney’s test *p* value: ∗ vs. MASL <0.05, ° vs. MASH-F01 < 0.05, ^∧^ vs. MASH-F2<0.05. § Mann-Whitney’s test *p* value T2D vs. noT2D < 0.05. TG, triglycerides.

### Glucose production is increased with MASLD severity, IR, and lipolysis

**Figure 1:**
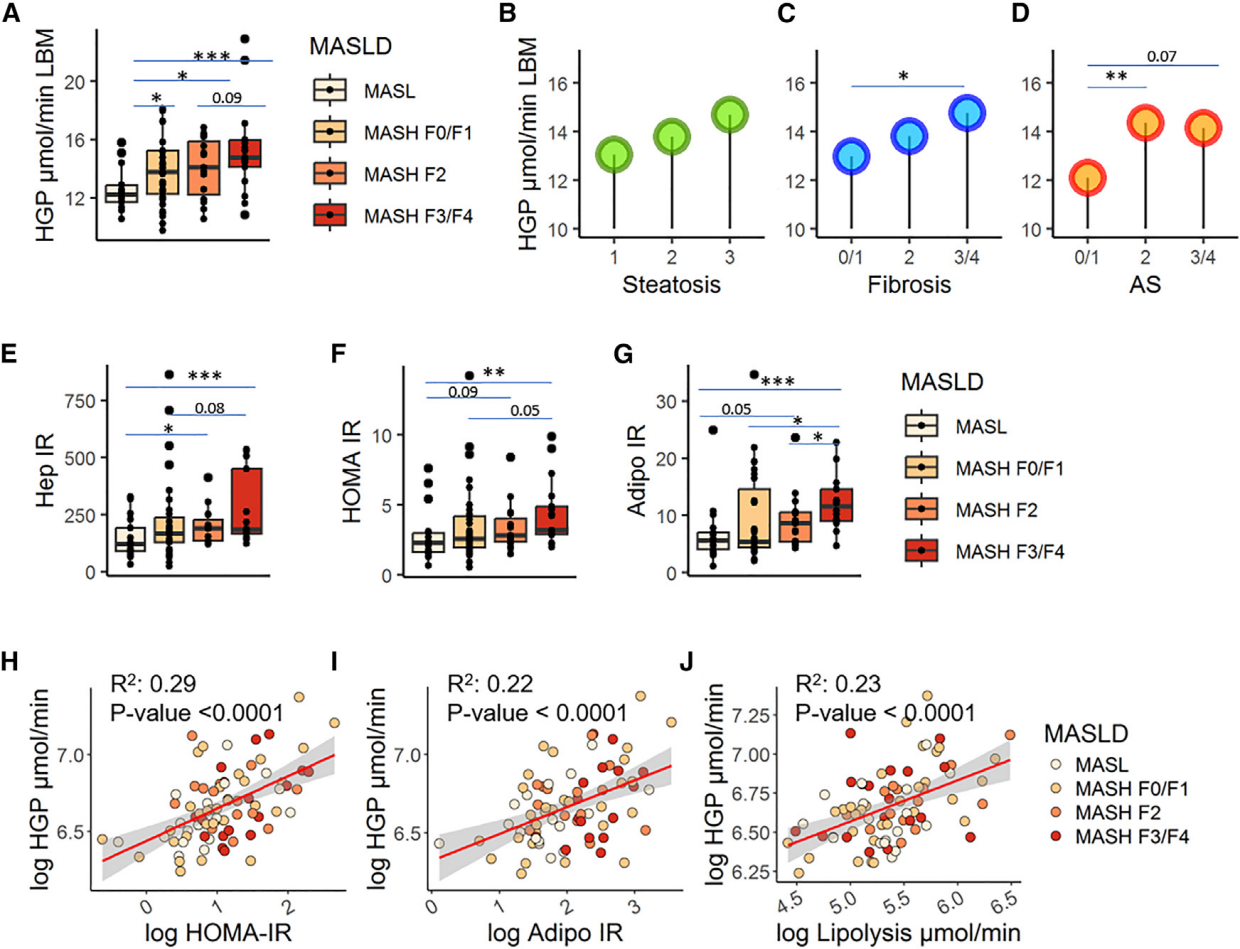
Glucose production, measured using stable isotope tracers, and insulin resistance in the EPoS-flux group (*n* = 80) In (A), boxplot of HGP grouping individuals according to MASL/MASH severity. In (B), (C), and (D), average HGP (mean) was reported considering the degrees of steatosis, fibrosis, and activity score (AS, inflammation + ballooning), respectively. In (E), (F), and (G), boxplots of insulin resistance in liver (Hep-IR, measured as μmol/min LBM ∗ mU/L), whole-body (HOMA-IR), and adipose tissue (Adipo-IR) in MASL/MASH groups, respectively. In (H), (I), and (J), linear associations of HGP with HOMA-IR, Adipo-IR, and lipolysis, respectively. The linear regression lines are colored in red while the area in gray indicates confidence interval at level 0.95. Dots were colored according to MASL/MASH severity. Mann-Whitney’s *p* value ∗<0.05, ∗∗<0.01, ∗∗∗ <0.001.

In the EPoS-flux group, tracer-measured HGP was significantly higher in subjects with MASH compared to MASL, with an increasing trend from MASL to MASH-F0/F1 to MASH-F2 to MASH-F3/F4 (Figure 1A, Kruskal-Wallis *p* value = 0.01). Increased HGP was not related to the degree of steatosis, but was significantly higher in those with fibrosis F3/F4 vs. F0/F1 and in those with activity score AS ≥ 2 (Figures 1B, 1C, and 1D), i.e., those with ballooning and inflammation, confirming that it is not the amount of hepatic lipids that serves as a marker of dysregulated hepatic glucose metabolism,^19^ but rather the degree of inflammation and fibrosis. Considering IR, Hep-IR showed a stepwise trend from MASL to MASH F0/F1 to F2 to F3/F4 (Figure 1E), indicating impaired insulin suppression of HGP. Similar trends were observed for the homeostatic model assessment for IR (HOMA-IR, Figure 1F) and for the adipose tissue IR index (Adipo-IR, Figure 1G) that also increased in a stepwise trend in individuals with MASH and moderate-to-advanced fibrosis compared to MASL and were positively associated with the absolute rates of HGP (Figures 1H and 1I). Moreover, adipose tissue lipolysis, measured by labeled glycerol infusion, was strongly correlated with HGP (Figure 1J), indicating the association between the elevated glucose production and the increased availability of glucogenic and energy substrates i.e., glycerol and free fatty acid (FFA), that occurs with the worsening of IR and the progression of MASLD to a more severe form. Indeed, higher FFA concentrations were observed in MASH-F3/F4 vs. MASL and their significant correlation with the plasma concentrations of β-hydroxybutyrate, a product of the hepatic fatty acid oxidation, indicates increased FFA uptake and oxidation by the liver (Figure S2).

### Genome-scale metabolic models for the study of intrahepatic glucose metabolism in subjects with MASH

**Figure 2:**
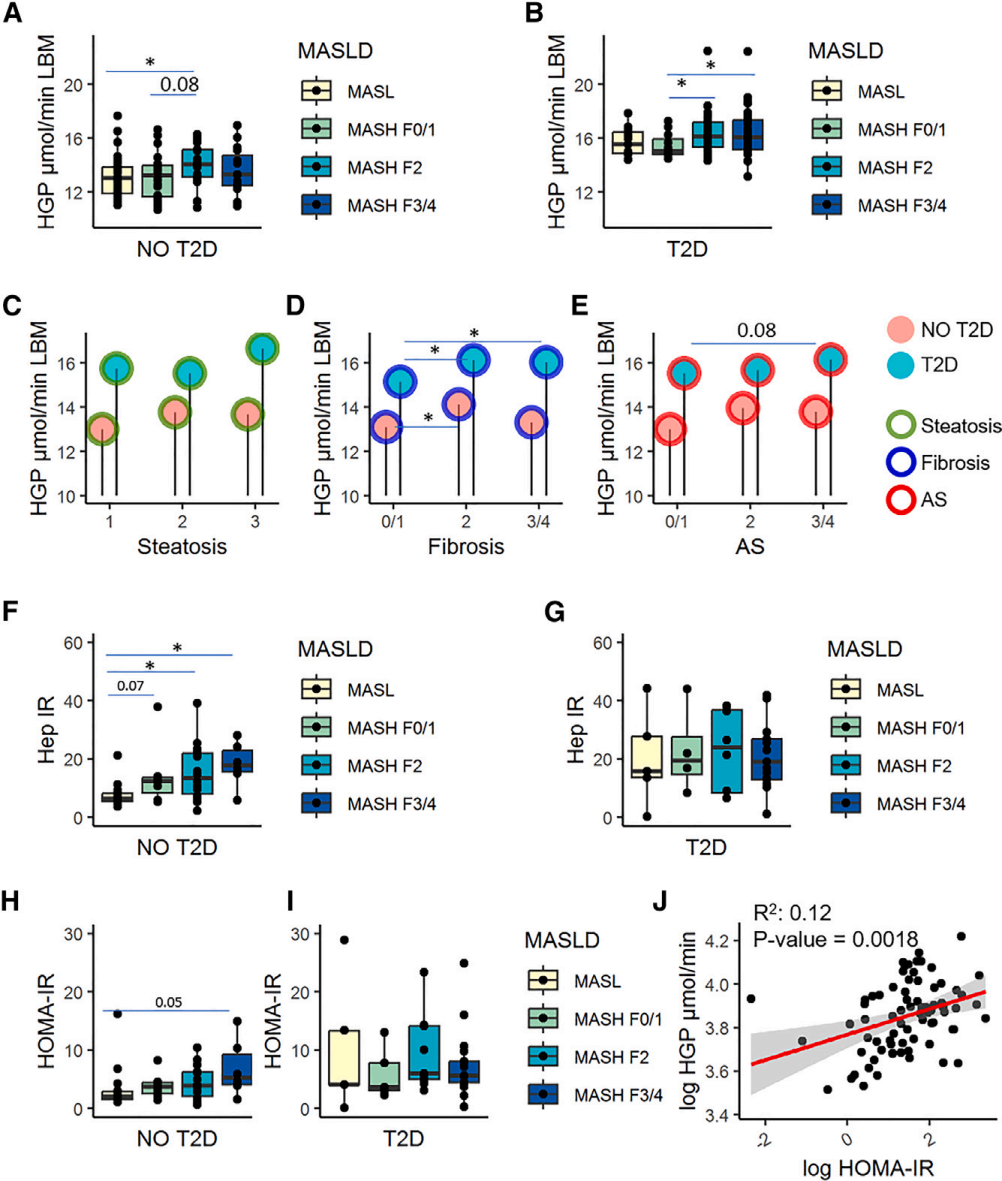
Glucose production, estimated using genome-scale metabolic models, and insulin resistance in the EPoS-transcriptomics subgroup (*n* = 206) In (A) and (B), boxplot of HGP in individuals without and with T2D, respectively. In (C), (D), and (E), average HGP (mean) was reported considering the degrees of steatosis (green), fibrosis (blue), and activity score (AS, inflammation + ballooning, red) in individuals with and without T2D, respectively. In (F), (G), (H), and (I), boxplots of hepatic insulin resistance (Hep-IR, as mmol/h LBM ∗ mU/L) and HOMA-IR in individuals without and with T2D, respectively. In (H) and (I), boxplots of whole-body insulin resistance (HOMA-IR) in individuals without and with T2D, respectively. In the boxplots, subjects were grouped according to MASLD histological severity. Mann-Whitney’s *p* value ∗<0.05, ∗∗<0.01, ∗∗∗ <0.001. In (J), linear associations of HGP with HOMA-IR were reported. The linear regression line is colored in red while the area in gray indicates confidence interval at level 0.95.

Although tracer kinetics is the gold standard for the measurement of metabolic fluxes in humans, other models have been recently proposed, like genome-scale metabolic models, that can be used as scaffolds to integrate multiple data.^20^ These models have been used and validated for estimating intrahepatic fluxes for hepatic lipid metabolism but never applied to the study of hepatic glucose metabolism in humans.^5^ In the EPoS-transcriptomics group (*n* = 206, Table 2), GEMs were constructed using individual gene expression and clinical data to estimate intrahepatic glucose fluxes that were then validated against tracer-measured fluxes, as reported later and detailed in the STAR Methods. HGP and Hep-IR, obtained using GEMs, showed the same trends observed in EPoS-flux group using tracer infusion (Figures 1 and 2). The EPoS-transcriptomics group includes subjects with and without T2D. Individuals without T2D showed similar characteristics in the EPoS-transcriptomics and the EPoS-flux group in terms of BMI, weight, liver enzymes, and glucose and insulin concentrations, while age and gender were slightly different (Figure S1B; Table S1). In individuals with T2D, HGP (Figures 2A and 2B) and Hep-IR (Figures 2F and 2G) were higher than in those without T2D, as expected. HGP increased with worsening of fibrosis and activity score (Figures 2D and 2E), but not with the degree of steatosis (Figure 2C), regardless of T2D. Hep-IR showed a stepwise trend in individuals without T2D (Figure 2F) as in tracer studies in Figure 1, while in the group with diabetes, Hep-IR was increased vs. subjects without T2D similarly in all MASH groups (Figure 2G). The same trend was observed for HOMA-IR (Figures 2H and 2I), although less significant, and the association between HOMA-IR and HGP was still present in the EPoS-transcriptomics cohort (Figure 2J).

**Figure 3:**
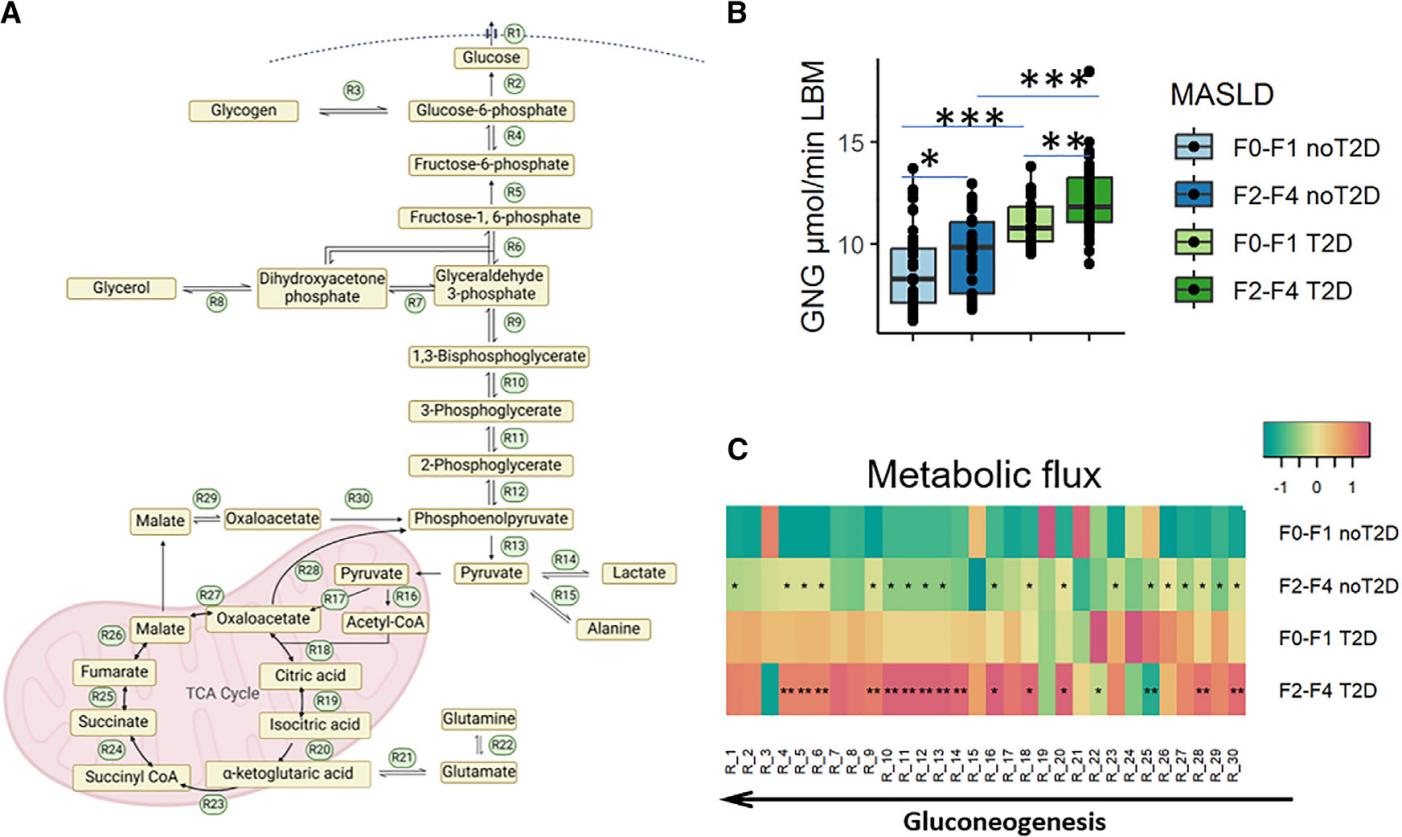
Gluconeogenesis is increased in individuals with severe fibrosis independently of T2D in the EPoS-transcriptomics group (*n* = 206) In (A), the schematic representation of fasting hepatic glucose metabolism, focusing on gluconeogenesis pathway and TCA cycle. In (B), the contribution of the gluconeogenesis flux to HGP expressed in μmol/min kg of LBM (GNG), estimated by genome-scale metabolic models in the EPoS-transcriptomics group. Individuals were grouped according to fibrosis stage and presence of T2D. Mann-Whitney’s test *p* values: ∗<0.05, ∗∗<0.01, ∗∗∗<0.001. In (C), heatmap of the estimated fluxes through the metabolic reactions in the subnetwork of (A). Each reaction in (A) is listed on the horizontal axis of the heatmap. Individuals were grouped according to fibrosis stage and T2D. Fluxes were scaled to zero mean and unit variance and reported as median within the groups. Mann-Whitney’s test *p* values vs. F0/F1: ∗< 0.1, ∗∗<0.05 after false discovery rate (FDR) correction, in diabetic and non-diabetic subgroups, respectively.

A major advantage of GEMs is the estimation of intracellular glucose fluxes (Figure 3A), which were different in individuals with vs. without severe fibrosis and T2D (Figure S3). Gluconeogenesis was higher in subjects with T2D, as expected,^5^ but the main finding here is the higher fluxes in MASH F2–F4 vs. F0/F1 particularly in those with T2D (Figures 3B, 3C, and S4) that were associated also with hepatic inflammation and fibrosis (Figure S4 D–G). Moreover, intracellular metabolic fluxes (see system depicted in Figure 3A) were higher in individuals with F2–F4 fibrosis compared to those with F0-F1 fibrosis (Figure 3C), especially in presence of T2D (Figure 3C), and correlated with the uptake of glucogenic precursors (Figure S4C).

### Alterations in hepatic gene expression involved in glucose and insulin metabolism in MASH with moderate-to-advanced fibrosis

The differences in the hepatic gene expressions among the MASL/MASH groups of the EPoS-transcriptomics group, encoded in GEMs, were milder (Figure 3A) compared to the differences in the metabolic fluxes (Figures 3C and S5). The expression of phosphoenolpyruvate carboxykinase-2 (R28) and α-ketoglutarate dehydrogenase (R23) was lower in F2–F4 with respect to F0-F1, regardless of the presence of T2D (Figure 3A), while other genes involved in the tricarboxylic acid (TCA) cycle were downregulated only in F2–F4 with T2D (Figure S5). On the contrary, both the expression of L-lactate dehydrogenase (R14) and fructose-biphosphatase-1 (R5) and the relative fluxes were significantly increased in individuals with moderate-to-advanced fibrosis and T2D (Figure S5).

**Figure 4:**
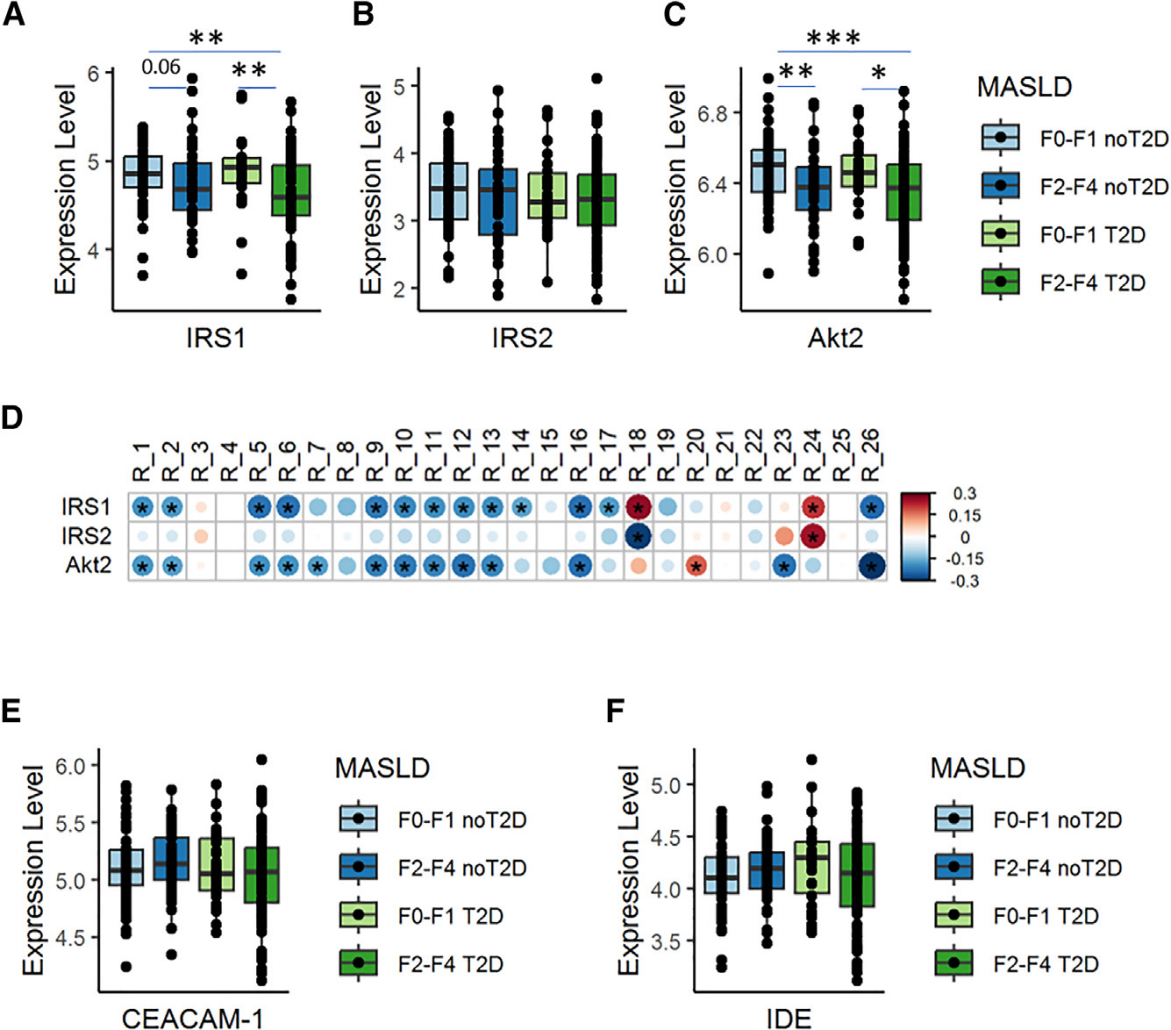
Hepatic expression of genes involved in insulin action but not insulin clearance is associated to advanced fibrosis, regardless of the presence of T2D in the EPoS-transcriptomics group (*n* = 206) In (A), (B), and (C), expression levels of IRS1, IRS2, and Akt2. Individuals were grouped according to fibrosis stage and presence of T2D. In (D), Spearman correlation matrix of the predicted fluxes through the system depicted in Figure 3A in the EPoS-transcriptomics cohort and the hepatic expression of the genes IRS1, IRS2, and Akt2. Significant correlation (*p* value < 0.05) were marked with ∗. In (E) and (F), expression levels of CEACAM-1 and IDE, according to the presence of advanced fibrosis and T2D. In the boxplots, Mann-Whitney’s test *p* values: ∗<0.05, ∗∗<0.01, ∗∗∗<0.001.

The expression of genes involved in insulin action and insulin clearance, not used for GEM reconstruction and thus not directly related to their predicted fluxes, was investigated. The hepatic expressions of insulin receptor substrates, like IRS1, IRS2, and AKT2, fundamental in glucose homeostasis,^21^ were significantly decreased in F2–F4 with respect to those with less severe forms of MASLD, in both diabetic and non-diabetic groups (Figures 4A–4C), consistently with the increased Hep-IR observed in Figures 1E and 2F. Moreover, their expression was inversely correlated with the intracellular glucose fluxes (Figure 4D).

Given that hepatic insulin clearance is decreased in MASLD,^22^ we also investigated genes involved in hepatic insulin metabolism, i.e., carcinoembryonic antigen-related cell adhesion molecule 1, CEACAM-1, involved in insulin internalization and insulin-degrading enzyme (IDE), but their expression was not altered with fibrosis stage or the presence of T2D (Figures 4E and 4F), nor with BMI.

### Validation of genome-scale metabolic models versus tracer models for glucose fluxes

GEMs were not previously used for the quantification of intrahepatic glucose fluxes, thus a validation of these estimates against gold standard methods is crucial to ensure their reliability. The validation was performed first by comparing the data of the individuals with glucose fluxes measured by both tracer infusion and personalized GEMs (*n* = 12) showing good correlation (r = 0.40, *p* = 0.03) and agreement (Figures S6A and S6B). Then, HGP and gluconeogenesis obtained with GEMs were validated in an independent cohort, the “GNG cohort,” which included individuals with MASLD, with and without T2D, sharing characteristics similar to the “EPoS cohort” in terms of age, gender, weight, BMI, alanine aminotransferase (ALT), and aspartate aminotransferase (AST) (Figure S6C). A significant correlation was observed between the fluxes measured by tracers and GEMs (rho = 0.32 and 0.25 for HGP and gluconeogenesis, respectively, *p* value <0.001, Figures S6D and S6E).

## Discussion

The regulation of hepatic glucose metabolism is complex and depends on several interdependent factors, both hepatic and extrahepatic.^23^^,^^24^ The present study explores the alterations in hepatic glucose metabolism across the full spectrum of MASLD, i.e., from MASL to MASH with different degrees of fibrosis in individuals with and without T2D. Compared to previous studies that measured glucose fluxes in subjects with different degrees of steatosis (MASLD),^3^^,^^5^^,^^19^^,^^25^ this study investigated HGP and gluconeogenesis in a large cohort of individuals with biopsy-proven MASL/MASH (EPoS cohort) and its relationship with the components of liver histology, with and without the presence of T2D.

We found that individuals with MASH and moderate-to-advanced fibrosis (F2 or higher) had higher glucose production than subjects with isolated steatosis (MASL) and that HGP increased also in the presence of hepatic inflammation and ballooning (activity score), but not steatosis (Figure 1). Furthermore, the increase in gluconeogenesis shown by the analysis of GEMs (Figures 3 and S4) and its strong correlation with HGP (rho = 0.58, *p* value <0.001, Figure S6F) points to gluconeogenesis as the major culprit of HGP. Higher HGP and gluconeogenesis were observed in MASH and moderate-to-advanced fibrosis regardless of the presence of T2D, but worse in those with diabetes. While it was known that subjects with T2D have higher gluconeogenesis and impaired suppression of glycogen breakdown,^26^^,^^27^^,^^28^^,^^29^ what was not known was that the presence of MASH and moderate-to-advanced fibrosis was associated with increased fasting glucose production driven by gluconeogenesis even in subjects without T2D, while no significant association was found with steatosis, confirming our previous report.^19^

The presence of excess HGP and Hep-IR has been previously reported in subjects with hepatic steatosis,^3^^,^^13^^,^^14^^,^^30^^,^^31^ and we showed here that having MASH and moderate-to-advanced fibrosis is associated with much higher hepatic IR than isolated steatosis, and glucose fluxes increase along with the severity of fibrosis (Figures 1E, 2F, and 2G).

It is known that individuals with obesity and/or T2D have higher prevalence of MASH and moderate-to-advanced fibrosis^9^^,^^10^ and having MASLD increases the risk of developing T2D.^6^ Excessive fat accumulation, in particular abdominal fat, has been found associated with the severity of MASLD/MASH and both visceral and hepatic fat are associated with increased adipose tissue and hepatic IR.^4^^,^^19^^,^^30^^,^^32^ Subjects with MASH of the EPoS cohort had high adipose tissue IR and increased lipolysis (Figure 1), resulting in high release of glycerol and FFA into the bloodstream. Splanchnic exchange of fuel substrates was found increased in subjects with obesity and/or T2D,^27^^,^^33^^,^^34^ and correlated with hepatic fat.^35^^,^^36^ When adipose tissue is resistant to the antilipolytic effect of insulin, glycerol is released together with fatty acids during lipolysis that stimulate gluconeogenesis.^37^^,^^38^ Consistently, the strong correlation between HGP and lipolysis (Figure 1J), measured as glycerol rate of appearance that at steady state equals the rate of peripheral uptake, indicates glycerol as an important substrate for hepatic gluconeogenesis, as previously suggested.^5^^,^^35^ The high fatty acid and glycerol flux to the liver determines an increase in hepatic acetyl CoA concentrations and in pyruvate carboxylase activity resulting in increased conversion of pyruvate to glucose.^39^ Thus, the putative mechanism responsible for the enhancement of fasting HGP and gluconeogenesis appears to be excess glucogenic substrates, like glycerol, lactate, and amino acids, not surprising given that MASH is a catabolic state.^40^ Also FFAs released during lipolysis play an important role since they are used not only for triglycerides synthesis^31^^,^^41^ but also as energy substrate for gluconeogenesis.^25^ The strong correlation between plasma FFA and β-hydroxybutyrate (Figure S2) in the presence of high lipolysis (Figure 1J) suggests a contribution to increased gluconeogenesis. Taken together, these findings suggest that lipolysis and gluconeogenesis are drivers of elevated glucose output in MASH, especially in those with moderate-to-advanced fibrosis.

It is established that not only metabolic but also genetic factors contribute to the pathophysiology of MASLD.^42^^,^^43^ The EPoS cohort provided important information also on the expression of genes that regulate hepatic glucose metabolism and insulin action. The transcriptomic analysis of liver biopsies did not show major alterations in genes related to gluconeogenesis and TCA cycle, although most genes were downregulated in subjects with MASH and moderate-to-advanced fibrosis/cirrhosis (Figure S5), but in agreement with previous studies in humans.^31^ Moreover, no differences or a lower expression of genes like phosphoenolpyruvate carboxykinase or glucose-6-phosphatase, catalytic (G6PC) were found in mice treated with a high-fat diet vs. controls or in humans with obesity undergoing bariatric surgery, despite fasting hyperglycemia and increased hepatic glucose fluxes.^44^^,^^45^^,^^46^^,^^47^^,^^48^ On the other hand, other studies with human liver biopsies showed that such genes were increased in subjects with MASLD as compared with normal liver but with mild differences between MASH and isolated steatosis.^49^^,^^50^

Knowing that HGP is tightly regulated by insulin that acts in the liver by activating a signaling cascade,^23^^,^^51^ we examined the expression of genes involved in insulin action, like IRS-1, IRS-2, or AKT2. We found that individuals with MASH and moderate-to-advanced fibrosis advanced fibrosis showed a downregulation of these genes (Figure 4), independently of diabetes, in agreement with previous reports.^31^^,^^50^^,^^52^ The expression of these genes was inversely associated with the estimated intracellular glucose fluxes (Figure 4D). It is to be noted that these genes were not included in the gene-protein-reaction rules used to build the GEMs nor were they used in the estimation of intracellular fluxes and, thus, their association with the predicted fluxes can be considered non-trivial. Finally, considering that insulin clearance is often found decreased in MASLD,^22^^,^^32^^,^^53^ we also investigated the expression of the genes CEACAM-1 and IDE involved in hepatic insulin metabolism,^22^ which did not change with severity of MASH. Altogether, this suggests that the enhanced production of hepatic glucose in MASLD was not associated with major changes in the expression of gluconeogenesis genes, but rather with the high *de novo* glucose synthesis driven by precursor availability and hepatic IR due to impaired hepatic insulin action, although a reduced hepatic insulin uptake may contribute to increased HGP.

In conclusion, we believe that the results of this study advance previous knowledge, highlighting the dysregulation of glucose hepatic metabolism as a major metabolic defect in subjects with MASLD that worsens as subjects with isolated steatosis progress to MASH with moderate-to-advanced fibrosis. These results highlight that inflammation/ballooning and fibrosis, not steatosis, are the markers of dysregulated glucose fluxes. The putative mechanisms responsible for these alterations involves both hepatic and adipose tissue IR and the consequent excess glucogenic and energy substrates to the liver, resulting in increased glucose production. This indicates a catabolic state and explains, at least partially, the mechanisms for increased risk of T2D and hyperglycemia in subjects with MASH.

### Limitations of the study

This study investigated hepatic glucose metabolism across the full spectrum of MASL/MASH in a large number of histologically characterized individuals and it presents strengths and limitations. The measurement of glucose fluxes was obtained using stable isotope tracers infusion, which represent the gold standard for the measurement of glucose fluxes *in vivo* in humans,^54^ while GEMs were employed to evaluate intrahepatic fluxes,^5^^,^^20^ which were here validated vs. tracer studies. A further strength of the study is the inclusion of a relatively large group of individuals with liver biopsy spanning across the full spectrum of MASLD, with and without T2D, showing that glucose fluxes are increased with MASH and fibrosis but also related to diabetic hyperglycemia. While tracer infusion is the gold standard technique, the predictions of metabolic fluxes using GEMs can be highly sensitive to model assumptions and constraints. Moreover, GEMs are tuned using transcriptomics data and it is known that RNA levels do not directly reflect metabolic flux and, as observed here as well, may even run in opposite directions (Figure S5). To validate the results presented here, we compared GEMs and tracer-derived data showing good correlations for HGP and gluconeogenesis (Figure S6) both in the EPoS cohort and in an independent cohort. Nevertheless, the use of GEMs to estimate intrahepatic metabolic fluxes in this paper should not be considered as an alternative to more accurate and quantitative methods, like PINTA,^55^ but rather an easy-to-access way to integrate different data into a putative model for the study of intrahepatic mechanisms without tracer infusion, providing insights coherent with the tracer-based evidences. Another possible limitation arises from the assumptions of some parameters that were derived from individual clinical data using a previously published methodology,^56^ since direct measurements of metabolites’ exchange rates within the liver were not available (their measurement would have required invasive procedures, e.g., catheterization of artery and hepatic and/or portal vein). However, despite these assumptions, the agreement between tracer-based and GEM fluxes in the validation cohorts demonstrates that the method is effective.

## Resource availability

### Lead contact

Further information and requests for resources should be directed to and will be fulfilled by the lead contact, Amalia Gastaldelli (amalia.gastaldelli@cnr.it).

### Materials availability

This study did not generate unique reagents.

### Data and code availability

• RNA-seq data have been deposited in the NCBI GEO database under accession number GEO: GSE135251 and are publicly available as of the date of publication. Scripts for GSMM, contextualization, and data analysis can be downloaded from https://doi.org/10.5281/zenodo.13837420 or alternatively from https://github.com/Silvia410/GSMM_liver. The personalized GEMs (.mat files) of human-hepatocytes are available upon request.
• Any additional information required to reanalyze the data reported in this paper is available from the lead contact upon request.

## Acknowledgments

We would like to thank the EPoS (Elucidating Pathways of Steatohepatitis) investigators and individuals that participated in the study (epos-nafld.eu) and Demetrio Ciociaro, Elisabetta Spagnolo, and Elisa Ferrari for the technical support. This work was supported by the European Union’s Horizon 2020 Research Programme for the project EPOS (grant agreement no. 634413), the Innovative Medicines Initiative 2 Joint Undertaking for the project LITMUS (grant agreement no. 777377), and the Innovative Health Initiative Joint Undertaking for the project GRIPonMASH (grant agreement no. 101132946). Additional support for this study was provided by 10.13039/501100009708Novo Nordisk Foundation (grant agreement no. NNF20OC0063971) and the 10.13039/501100002341Research Council of Finland (grant agreement no. 333981). Graphical abstract has been created in BioRender (Sabatini, S. [2024] BioRender.com/s65e318).

## Author contributions

Conceptualization, A.G. and M.O.; methodology, P.S. and S.S.; investigation, F.C., S.P., T.H., A.D., O.G., and S.C.; resources, C.R., E.L., and O.V.; formal analysis, S.S.; writing – original draft, S.S. and A.G.; writing – review and editing, S.S., A.G., and M.O.; supervision, A.G., M.O., Q.M.A., E.B., and G.M.; funding acquisition, A.G., M.O., Q.M.A., E.B., and G.M.

All authors critically revised the manuscript for intellectual content and approved the final manuscript. A.G. and M.O. are the guarantors of this work and, as such, had full access to all the data in the study and take responsibility for the integrity of the data and the accuracy of the data analysis.

## Declaration of interests

G.M. reports consulting fees from Novo Nordisk, Fractyl Inc, and Recor Inc. She is also scientific advisor of Metadeq Inc, Keyron Ltd, GHP Scientific Ltd, and Jemyll Ltd. G.M. reports receiving research grants from Metadeq Inc and Fractyl Inc. A.G. has served as a consultant for: Boehringer Ingelheim, Eli Lilly and Company, Metadeq Diagnostics, and Fractyl Health; has participated in advisory boards for Boehringer Ingelheim, Merck Sharp & Dohme, Novo Nordisk, Metadeq Diagnostics, and Pfizer; and has received speaker’s honorarium and other fees from Eli Lilly and Company, Merck Sharp & Dohme, and Novo Nordisk. Q.M.A. reports grants and/or personal fees from Allergan/Tobira, E3Bio, Eli Lilly & Company Ltd, Galmed, Genfit SA, Gilead, Grunthal, Imperial Innovations, Intercept Pharma Europe Ltd, Inventiva, Janssen, Kenes, MedImmune, NewGene, Pfizer Ltd, Raptor Pharma, Novartis Pharma AG, AbbVie, BMS, GSK, NGMBio, Madrigal, Servier, EcoR1, 89Bio, Altimmune, AstraZeneca, Axcella, Blade, BNN Cardio, Celgene, Cirius, CymaBay, Genentech, HistoIndex, Indalo, IQVIA,Metacrine, North Sea Therapeutics, Novo Nordisk, Poxel, Terns, Viking Therapeutics, Glympse Bio, and PathAI, outside the submitted work.

## STAR★Methods

### Key resources table

**Table:** 

REAGENT or RESOURCE	SOURCE	IDENTIFIER
**Chemicals, peptides, and recombinant proteins**		

D-Glucose (6,6-D2, 99%)	Cambridge Isotope Laboratories	CAT# DLM-349-PK
Glycerol (1,1,2,3,3-D5, 99%)	Cambridge Isotope Laboratories	CAT# DLM-1229-PK

**Deposited data**		

seq raw and analyzed data	Govaere et al.^18^	NCBI GEO repository: GEO: GSE135251

**Software and algorithms**		

R Statistical Software (version 4.0.5)	R Foundation for Statistical Computing, Vienna, Austria	https://cran.r-project.org/
MATLAB 2019b	Mathworks, Inc., Natick, MA, USA	https://se.mathworks.com/
Cobra toolbox v3.0	Heirendt et al.^57^	https://opencobra.github.io/
RAVEN suite 2.0	Wang et al.^58^	https://github.com/SysBioChalmers/RAVEN
Code for core analyses and generation of all main figures	Silvia Sabatini (silviasabatini@cnr.it); Zenodo	https://doi.org/10.5281/zenodo.13837420

### Experimental model and study participant details

#### EPoS cohort

In this study, we consider a subset of 274 individuals with liver biopsy from the EPoS-cohort,^59^ for which liver transcriptomics (EPoS-transcriptomics, *n* = 206) and tracer-based fluxes (EPoS-flux, *n* = 80) were collected (Figure S1). The liver biopsies’ samples obtained in this study were centrally scored by two expert liver pathologists according to the semiquantitative NASH-Clinical Research Network ‘NAFLD Activity Score’ (NAS).^60^ Fibrosis was scored from F0 to F4 (cirrhosis). For all subjects, alcohol consumption was within the limit of 30g/day for male and 20g/day for female at the time of recruitment. All of the patients are from European descent. All studies were approved by local and/or national Ethical Review Committees covering each participating center, with all patients providing informed consent for participation. All participant recruitment and informed consent processes were conducted in accordance with the World Medical Association Declaration of Helsinki 2018. The anthropometric and clinical characteristics of the EPoS-flux and the EPoS-transcriptomics groups are reported in Tables 1 and 2.

#### GNG cohort

Data from an additional independent cohort (GNG cohort) were used for validation of the glucose fluxes estimated by using genomes-cale metabolic modeling. The GNG cohort consisted of individuals with and without T2D (*n* = 57) spanning a wide range of obesity.^19^ In this cohort liver fat content was measured by magnetic resonance spectroscopy and glucose fluxes, i.e., HGP and gluconeogenesis, were measured by tracer infusion and ^2^H_2_O ingestion respectively, which is considered the gold standard tracer method.^19^ For GEM analysis we included only subjects with characteristics comparable to EPoS-transcriptomics, i.e., those having MRI liver fat >5% or fibrotic NASH index^61^ FNI ≥0.33 (*n* = 50). The study protocol was approved by the Institutional Review Board of the University of Texas Health Science Center at San Antonio, and informed written consent was obtained from each patient before participation. The anthropometric and clinical characteristics of the GNG cohort are reported in Table S2.

### Method details

#### Transcriptomics

Whole liver tissue RNA-Seq transcriptomic data were collected from biopsy’s samples and are publicly available in the NCBI GEO repository: GSE135251. A description of transcriptomic analysis used in this study is detailed in.^18^

#### Tracer-based fluxomics

Endogenous glucose production that is mainly hepatic (HGP, μmol/min) was measured by the kinetics of 6,6-^2^H_2_-glucose following a protocol previously described in details.^54^ The fractional contribution of gluconeogenesis to HGP was calculated as the product to precursor ratio and gluconeogenesis flux (GNG) was then calculated by the product of %GNG times HGP. The fluxes were normalized by lean body mass (LBM) since this allows to account for the variability due to different degrees of obesity.^17^ Adipose tissue lipolysis, i.e., rate of appearance of glycerol (μmol/min), by the kinetics U-^2^H_5_-glycerol. The two stable isotopes were co-infused for 2 h during fasting state.

### Quantification and statistical analysis

#### Body composition estimates

It is well established that total body endogenous glucose output variability is wide and is largely explained by the amount of lean mass (LBM).^17^ For subjects that do not have measurement of lean/fat mass, they were estimated using Hume’s formula.^62^ Muscle mass was calculated for each subject as 0.63 x LBM – 4.1 as suggested by Mardinoglu et al.^56^

#### Calculation of insulin resistance indexes

Indexes of insulin resistance were calculated as follows.

• Hepatic insulin resistance^19^^,^^54^ (Hep-IR) = HGP x Ins,
• Adipose tissue index (Adipo-IR) = FFA x Ins
• HOMA-IR = Glucose(mmol/l) x Ins(mU/l)

#### Genome-scale metabolic modeling and flux analysis

##### GEMs description

In this paper, GEMs were used to investigate hepatic glucose metabolism in fasting conditions across the full spectrum of MASLD. Personalized genome-scale metabolic models of human liver were initially developed by Sen et al.,^63^ mapping transcriptomic data of the 206 subjects from the EPoS-transcriptomics group into *iHepatocytes2322*^64^*,* used as a template model, and deriving individual constraints for the metabolic reaction fluxes. A detailed description of models’ development can be found in,^63^ and the GEMs were tested to carry out 256 metabolic tasks exhibited by human liver.^64^ However, the GEMs developed by Sen et al. in^63^ did not include glycogenolysis, that is an important contributor to HGP. Thus, the GEMs were modified to enable a net consumption of glycogen by introducing a new reaction for the total glycogen breakdown, as previously done in.^35^

##### Constraints on the exchange fluxes in the fasting state

During fasting, the liver is the main source of glucose production derived from both gluconeogenesis and glycogenolysis. Glucogenic substrates, like lactate, amino acids and glycerol, released by peripheral tissues, are taken up by the liver and directed to gluconeogenesis. At the same time, glycogen stored in the liver is broken down to produce glucose. Liver also takes up free fatty acids (FFA), released as well from adipocytes by lipolysis. Although they are not glucose precursors, FFA contribute to the formation of newly synthesized di- or tri-acylglycerols or are oxidized to produce energy for the TCA cycle or ketone bodies. Moreover, the increased production of hepatic acetyl-coA increases the activity of pyruvate carboxylase thus contributing to increased HGP and hepatic insulin resistance.^39^

Conditions like obesity, insulin resistance or T2D could alter the efflux of these substrates to the liver. Thus, we contextualized the GEMs on glucose metabolism by incorporating individual constraints on the exchange fluxes between the liver and peripheral tissues (such as muscle and adipose tissue) that release glucogenic/energy substrates during fasting. This methodology mirrors a previous approach suggested by Mardinoglu^56^ and for each model and substrate, we derived upper and lower bounds from the literature, based on body composition. Since, unlike the cohort of subjects studied in,^56^ the EPoS-transcriptomics group comprises subjects with and without T2D, we adapted the calculations to our context, taking into account the presence of diabetes. The recent review of Shah et al.^65^ retrieves from the literature evidence of the relative changes to glucose contribution from several precursors in the setting of T2DM with respect to metabolically healthy controls, i.e., 2-fold increase for lactate and glutamine and 1.5-fold increase for alanine. Such relative changes were used as multiplying factors for the estimates of lactate, alanine, glutamine and glycerol in subjects with BMI<30 and a diagnosis of T2D.

Constraints on the upper bound of the glycogenolytic flux were imposed according to the rates of glycogenolysis measured by tracers in subjects with or without T2D and BMI higher or lower than 27 kg/m^2^ and reported in.^27^ Additional constraints for the GEMs were imposed on exchange reactions to allow the uptake of other compounds necessary for the reactions, such as oxygen, phosphate, minerals, etc., and the production of lipids (‘HMR_0031’). The uptake of further metabolites, e.g., glucose, was blocked since not relevant in the fasting state for the study of glucose metabolism.

##### GEM reaction activity scores

Gene-protein-reaction (GPR) rules are logical expressions used in GEMs to link genes expression to reactions. To evaluate the transcriptomic control over liver metabolism, the reaction activity score was computed for each reaction and individual model, based on the expression of the genes encoding for catalyzing enzymes and on the relationship among them, expressed by the GPR rules: for reactions involving enzymes composed by different subunits, the reaction activity score was computed as the minimum of the expressions of the genes encoding the subunits, while in the case of reactions catalyzed by different enzyme isoforms, the reaction activity score was computed as the maximum of the expressions of the genes encoding the isoforms.

##### GEM flux estimation

The range of feasible fluxes through each reaction was computed using flux variability analysis (FVA). Reversible reactions were represented as two distinct and complementary forward reactions. To obtain a single value for each reaction, we considered the average between maximal and minimal possible fluxes. As objective function, we set the export of glucose, i.e., ‘HMR_9034’ or, equivalently R1 in Figure 3A, since we focused our interest on glucose metabolism in fasting conditions. Flux rates exceeding mean value of three times the standard deviation were considered outliers and excluded from the analysis. Linear programming and optimization were performed using ’ILOG-IBM CPLEX (version 128)’ solver. Simulations were performed using Cobra toolbox v3.0^57^ and RAVEN 2.0 suite.^58^ All the operations were performed in MATLAB 2019b (Mathworks, Inc., Natick, MA, USA). HGP was calculated as the mean value of fluxes range determined by FVA for the exchange reaction of glucose in the models (R1 in Figure 3). To calculate the separate contribution of glycogenolysis and gluconeogenesis to HGP, for each model flux balance analysis (FBA) was performed by setting HGP as objective function. The absolute contribution of glycogenolysis to HGP was computed by multiplying the rate of glycogen breakdown predicted by FBA for the associated Lagrange multiplier (shadow price), while gluconeogenesis was obtained by subtracting the contribution of glycogenolysis to the total HGP. The Lagrange multipliers associated to the optimization problem solved by FBA can be interpreted as the change in the objective function by relaxing a constraint by one unit. In this context, this represents the variation in HGP by consuming one more unit of glycogen. This method can be used also to calculate the separate contribution of the glucose precursors to gluconeogenesis. In the EPoS-transcriptomics group, Hep-IR was estimated as HGP x insulin in the subgroup of subjects that had insulin measurements available at the time of liver biopsy.

#### Validation of GEM-derived glucose fluxes vs. tracer-based models

GEM estimates of metabolic fluxes were doubly validated. First, we directly compared HGP estimated by GEMs and measured by tracers in the subgroup of subjects that had both liver transcriptomics and were studied with tracer infusion (*n* = 12). Then, we used GEMs parameters, derived from the ‘EpoS-transcriptomics’ group by matching the subjects according to sex, age, presence of T2D (yes/no), weight, BMI, ALT, AST, to estimate total HGP and the contribution derived from gluconeogenesis. The matching was done minimizing Euclidean distance between individuals belonging to different cohorts (Figure S6E). Fluxes experimentally measured by tracers or estimated with GEMs were compared using linear regression.

#### Statistical analysis

Subjects were grouped according to presence of MASH vs. MASL, T2D, and degree of liver fibrosis. Comparisons among two or more groups were performed by using Mann-Whitney’s or Kruskal-Wallis’ test, respectively. Homogeneity between EPoS-flux and EPoS-transcriptomics group was assessed by principal component analysis (PCA). All statistical analysis was performed using R Statistical Software (version 4.3.3). Data in tables were reported as mean ± standard error. Heatmaps were created reporting data as median within the groups of interest. Datasets were centered and scaled in the row direction to improve interpretability. Statistical significance in the heatmap was reported after adjusting for multiple comparison, using Benjamini–Hochberg procedure.

## Supplemental information

Document S1. Figures S1–S6 and Tables S1 and S2

Document S2. Article plus supplemental information
