# Supplementary material for: Hepatic glucose production rises with the histological severity of metabolic dysfunction-associated steatohepatitis
Source: Cell Rep Med. 2024 Nov 19;5(11):101820. doi: 10.1016/j.xcrm.2024.101820 (PMC11604487; doi:10.1016/j.xcrm.2024.101820)
Supplement: Document S1. Figures S1–S6 and Tables S1 and S2 [file mmc1.pdf]

Cell Reports Medicine, Volume *5*

Supplemental information

Hepatic glucose production rises with the histological severity of metabolic dysfunction-associated steatohepatitis

Silvia Sabatini, Partho Sen, Fabrizia Carli, Samantha Pezzica, Chiara Rosso, Erminia Lembo, Ornella Verrastro, Ann Daly, Olivier Govaere, Simon Cockell, Tuulia Hyötyläinen, Geltrude Mingrone, Elisabetta Bugianesi, Quentin M. Anstee, Matej Orešič, and Amalia Gastaldelli

Supplementary Tables

Table S1 – Clinical characteristics of subjects without T2D in the EPoS-flux and EPoS-transcriptomics groups. Related to Fig. 1 and 2.

	EPoS-flux		EPoS-transcriptomics		P.value
	N	Mean±SE	N	Mean±SE	
Age (y)	80	41.24±1.32	97	50.4±1.43	<0.001
Sex (M/F)	80	67/13	97	63/34	0.009
Weight (kg)	80	90.31±2.79	97	87.42±1.73	0.47
BMI (kg/m^2)	80	29.91±0.8	97	30.35±0.58	0.06
ALT (iU/L)	80	72.14±4.4	96	71.6±5.35	0.45
AST (iU/L)	79	38.34±1.77	92	43.37±2.72	0.28
Glucose (mg/dL)	80	95.7±1.23	54	97.21±3.04	0.56
Insulin (mU/L)	80	14.83±1.04	42	17.24±2.7	0.66

Mann-Whitney's test p-value was reported.

Table S2 – Clinical characteristics of the GNG cohort. Related to STAR Methods.

Name	N	GNG Cohort
Age (y)	57	51.05±1.5
Sex (M/F)	57	37/20
T2D	57	43/14
Liver Fat (%)	57	17.37±1.63
FNI	48	0.39±0.04
Weight (kg)	57	80.81±2.11
BMI (kg/m^2)	57	27.82±0.55
ALT (iU/L)	49	26.37±1.91
AST (iU/L)	49	22.35±1.38
Glucose (mg/dL)	56	159.14±6.5
Insulin (mU/L)	57	82.39±7.08

Supplementary Figures

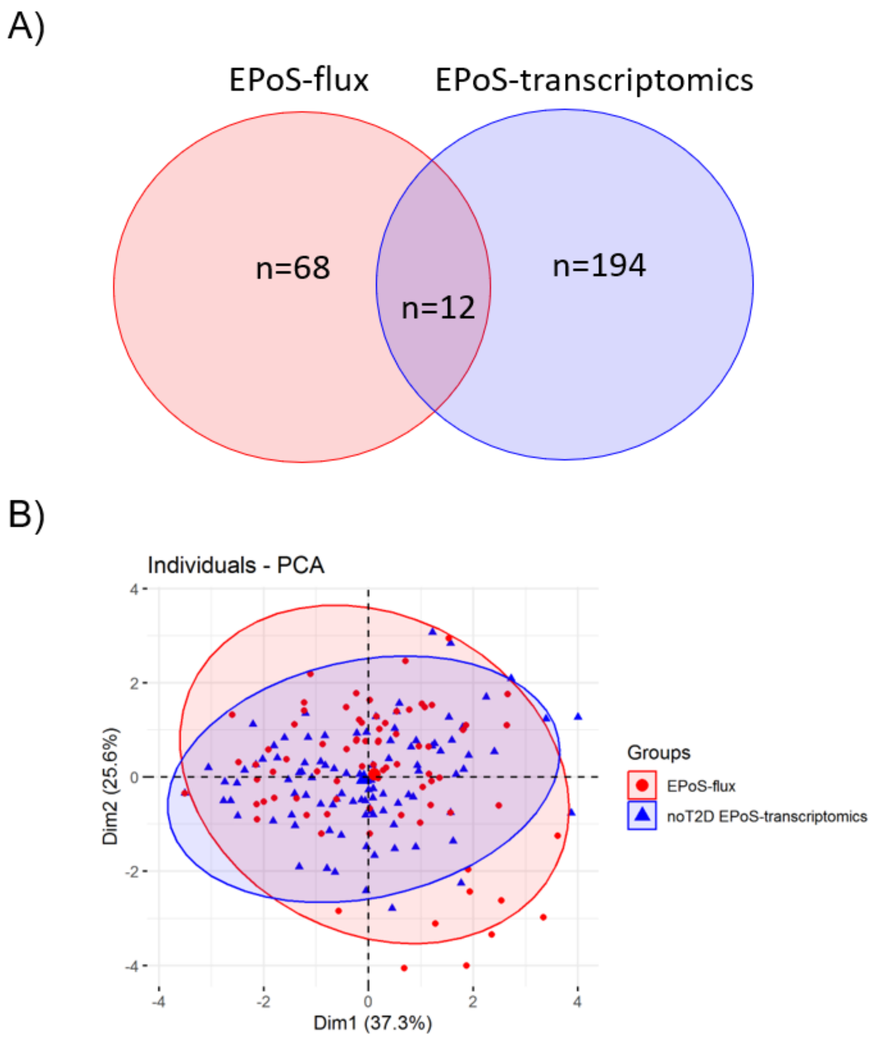

Figure S1. Distribution of subjects in the EPoS cohort. Related to Fig. 1 and 2. A) Venn diagram of individuals from the EPoS cohort belonging to the EPoS-flux and/or the EPoS-transcriptomics groups. B) Principal Component Analysis of individuals in the EPoS-flux group and individuals without T2D of the EPoS-transcriptomics group, according to age, sex, weight, BMI, ALT, AST.

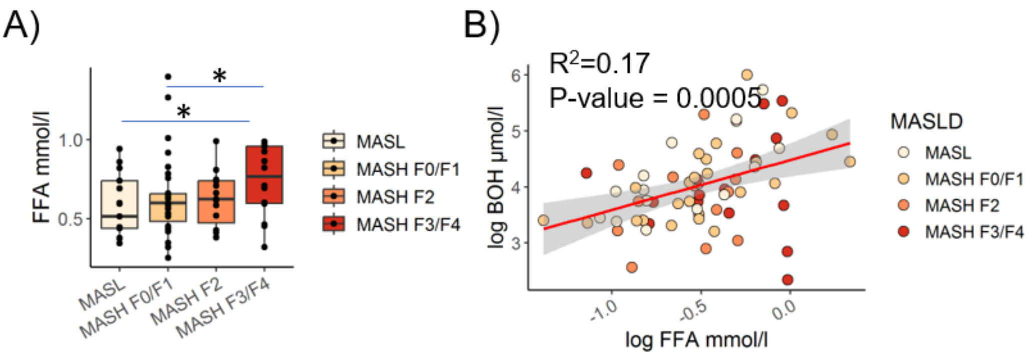

Figure S2. FFA and β-hydroxybutyrate in the EPoS-flux group. Related to Fig. 1. Panel A, FFA concentration grouping the individuals in the EPoS-flux group according to MASLD histological severity. Panel B, linear association of FFA with β-hydroxybutyrate. The linear regression lines are colored in red while the area in grey indicates confidence interval at level 0.95. Mann-Whitney's p-value *<0.05.

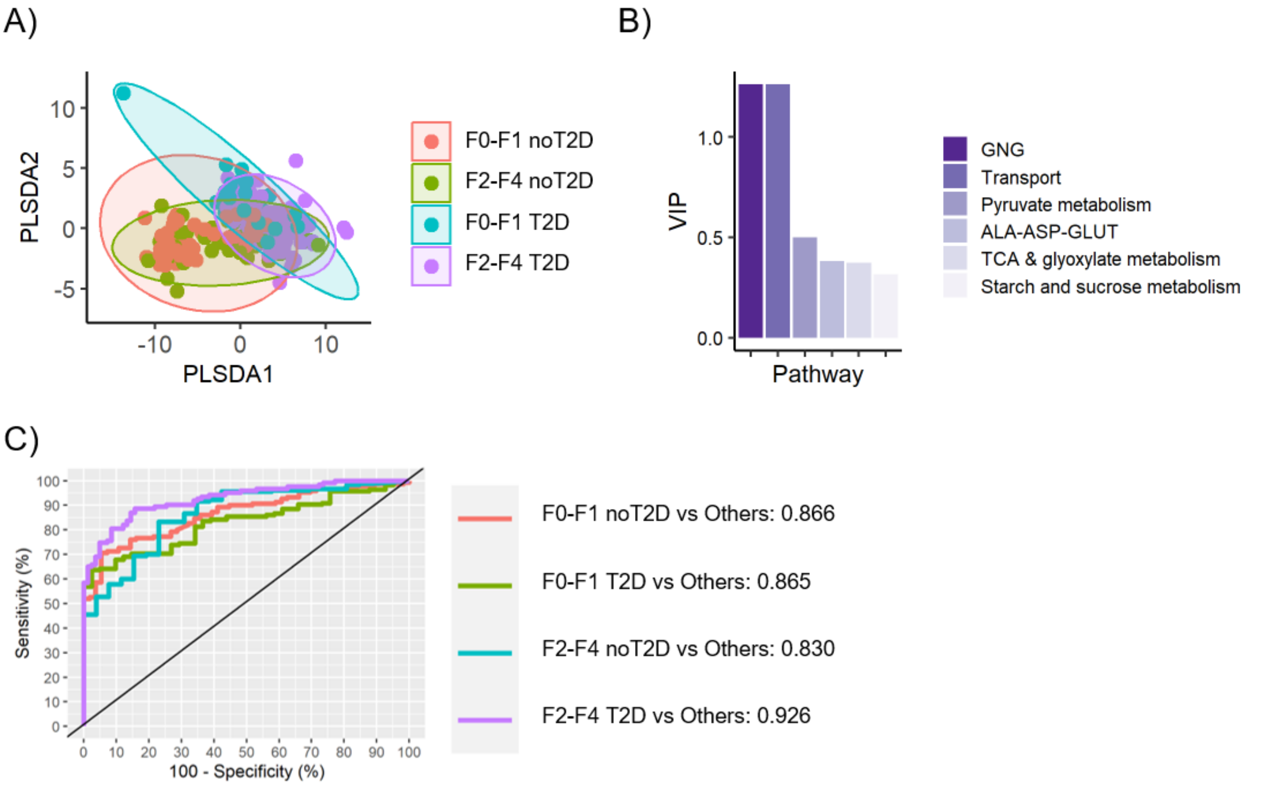

Figure S3. Least Square Discriminant Analysis of estimated fluxes in the EPoS-transcriptomics group. Related to Fig. 3. Panel A: PLS-DA score plot according to the severity of fibrosis and the presence of T2D. Panel B: average VIP score within the considered pathways. Panel C: performance of PLS-DA model for the classification of each class vs all the others, measured by ROC curves and relative areas under the curve (AUC).

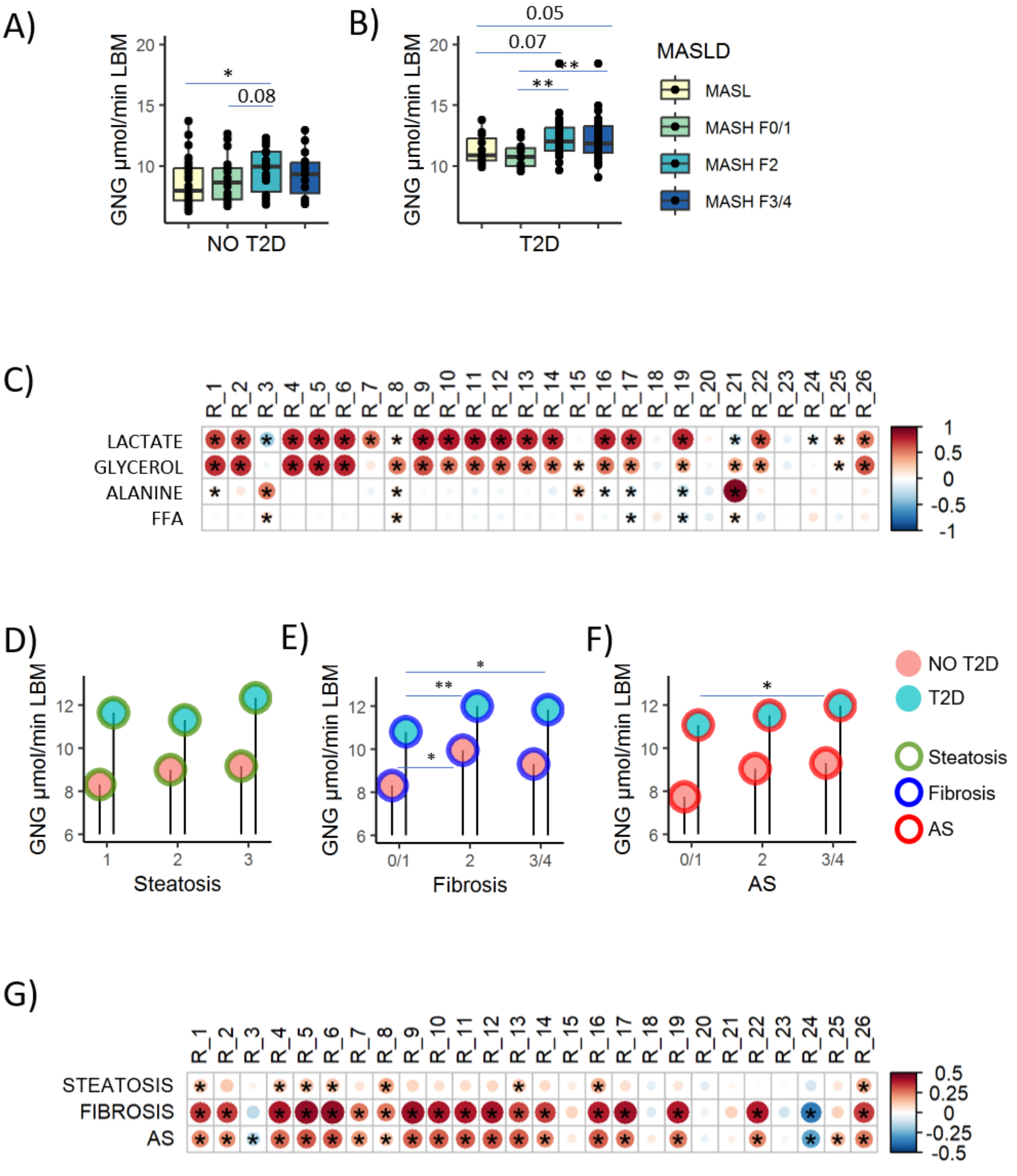

Figure S4. Gluconeogenesis contribution to HGP, estimated using genome-scale metabolic models in the EPoS-transcriptomics group. Related to Fig. 3. In panel A, B boxplot of glucose production derived from gluconeogenesis (GNG) in individuals without and with T2D, respectively. Subjects were grouped according to MASLD histological severity. In panel C, spearman correlation matrix between the predicted fluxes through the system depicted in Fig. 3A and the constraints (upper bounds) on the uptake of glucogenic/energy substrates (lactate, glycerol, alanine and FFA) during fasting, determined integrating clinical data of the subjects. In panel D, E, F average GNG was reported considering the degrees of steatosis (green), fibrosis (blue) and activity score (inflammation + ballooning, red) in individuals with and without T2D, respectively. In panel G, spearman correlation matrix between the predicted fluxes

through the system depicted in Fig. 3A and the degrees of livers steatosis, fibrosis and activity score. Pairwise comparison Mann-Whitney's p-values <0.05, <0.01, <0.001 in panel A, B, D, E, F were marked with *, ** or ***, respectively. Significant correlation (p-value <0.05) in panel C and G were marked with *.

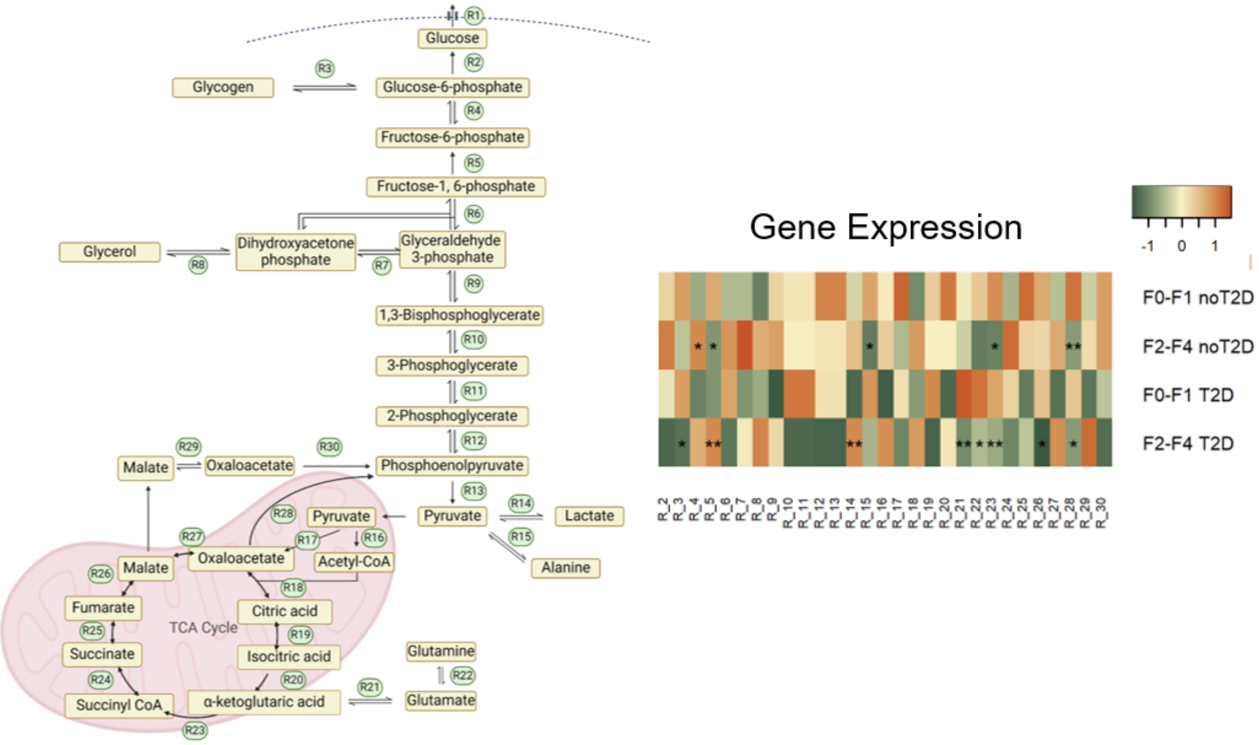

Figure S5. Gene expression in the EPoS-transcriptomics group. Related to Fig. 3. Heatmap of the reaction activity scores retrieved from gene expression data associated to the reactions depicted in Fig. 3A. Each reaction in 3A is listed on the horizontal axis of the heatmap. Individuals were grouped according to the severity of fibrosis and presence of T2D. Features were scaled to zero mean and unit variance and reported as median within the groups; Mann-Whitney's test p-values vs F0/F1: *< 0.1, **<0.05 after FDR correction, in diabetic and non-diabetic subgroups, respectively.

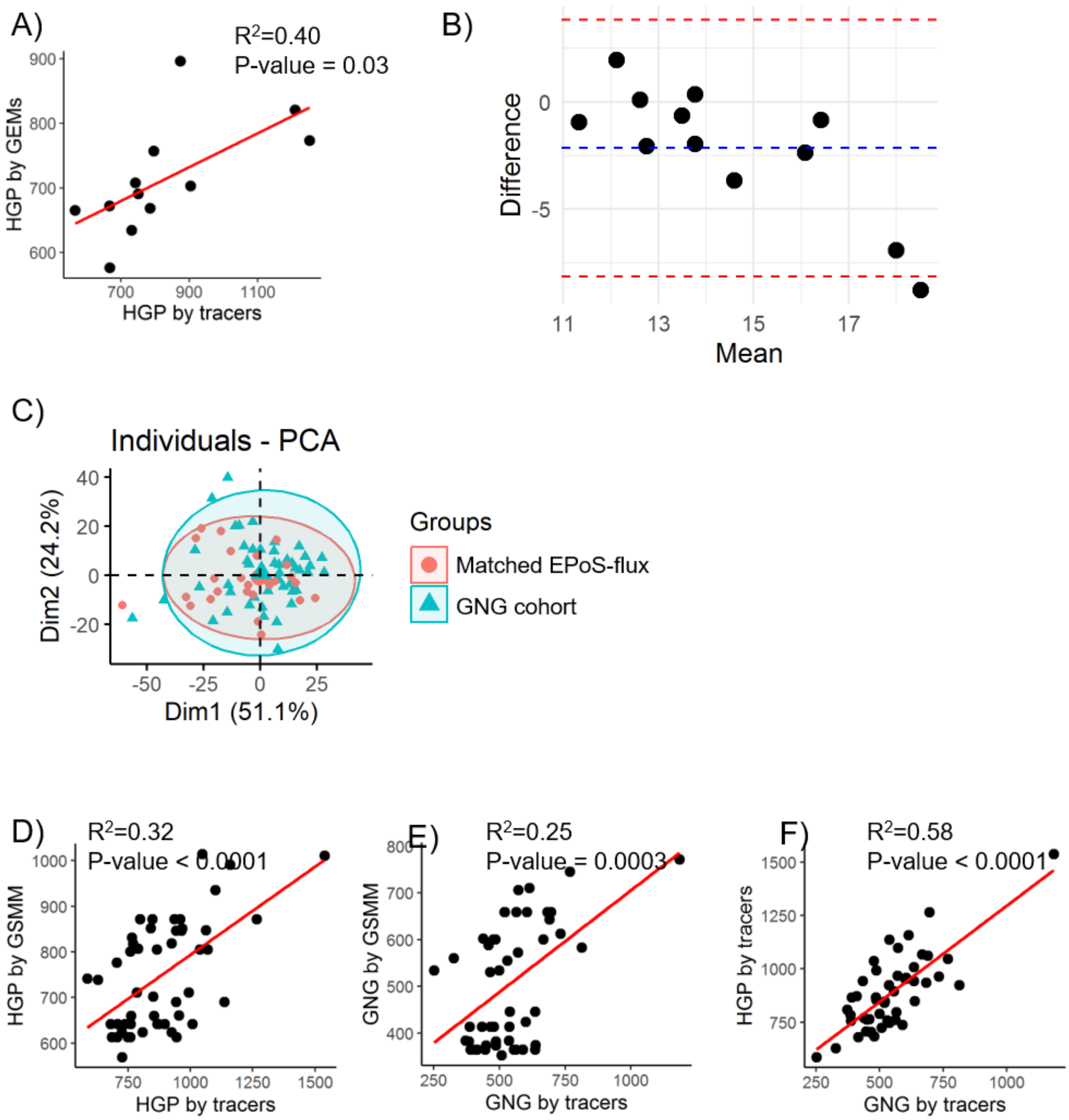

Figure S6. Validation of glucose fluxes estimated in the EPoS-transcriptomics group using genome-scale metabolic models versus tracer-based measurements. Related to STAR Methods. Panel A and B: agreement between glucose production obtained with GEMs vs stable isotope tracer infusion in the 12 EPoS subjects with both measurements, evaluated through linear association and Bland-Altman analysis, respectively. Panel C: Principal Component Analysis of the individuals in the GNG cohort and those in the EPoS-flux group that were matched 1:1 according to age, sex, weight, BMI, ALT, AST. Panel D and E show the linear association of HGP and gluconeogenesis (GNG) fluxes () estimated by the GEMs vs those measured by tracers in the GNG cohort. Panel E shows the strong correlation between HGP and GNG fluxes measured using stable isotope tracers in the GNG cohort. All fluxes are expressed in µmol/min.
